# Supplementary material for: 1q amplification and PHF19 expressing high-risk cells are associated with relapsed/refractory multiple myeloma
Source: Nat Commun. 2024 May 16;15:4144. doi: 10.1038/s41467-024-48327-9 (PMC11099140; doi:10.1038/s41467-024-48327-9)
Supplement: Supplementary file 1 — Supplementary Information [file 41467_2024_48327_MOESM1_ESM.pdf]

SUPPLEMENTARY MATERIALS: 1q amplification and PHF19 expressing high-risk cells are associated with relapsed/refractory multiple myeloma

AUTHOR LIST:

Travis S. Johnson[1,2,3,4], Parvathi Sudha[5], Enze Liu[5], Nathan Becker[5], Sylvia Robertson[2], Patrick Blaney[6], Gareth Morgan[6], Vivek S. Chopra[7], Cedric Dos Santos[7], Michael Nixon[8], Kun Huang[1,3,4], Attaya Suvannasankha[5,9], Mohammad Abu Zaid[5], Rafat Abonour[5], Brian A. Walker[4,5]

AFFILIATIONS:

[1]Department of Biostatistics and Health Data Science, School of Medicine, Indiana University, Indianapolis, IN, USA

[2]Indiana Biosciences Research Institute, Indianapolis, IN, USA

[3]Melvin and Bren Simon Comprehensive Cancer Center, Experimental and Developmental Therapeutics, School of Medicine, Indiana University, Indianapolis, IN, USA

[4]Center for Computational Biology and Bioinformatics, School of Medicine, Indiana University, Indianapolis, IN, USA

[5]Melvin and Bren Simon Comprehensive Cancer Center, Division of Hematology and Oncology, School of Medicine, Indiana University, Indianapolis, IN, USA

[6]Perlmutter Cancer Center, Langone Health, New York University, New York, NY, USA

[7]Genentech Inc., South San Francisco, CA, USA

[8]Roche Inc., Indianapolis, IN, USA

[9]Roudebush VAMC, Indianapolis, IN, USA

Correspondence: Brian A. Walker, C310 Walther Hall, 980 W Walnut St, Indiana University, Indianapolis, IN, 46202. bw75@iu.edu
*Corresponding Author Email: bw75@iu.edu

ABBREVIATIONS: 30 Abbreviations
SUPPLEMENTARY FIGURES: 11 Figures
SUPPLEMENTARY TABLES: 12 Tables
SUPPLEMENTARY FILES: 6 Files

ABBREVIATIONS
CNV: Copy number variant
SNP: Single nucleotide polymorphism
VAF: Variant Allele Frequency
dbSNP: The Single Nucleotide Polymorphism Database
RS: Reference single nucleotide polymorphism ID in dbSNP
WGS: Whole genome sequencing
CPM: Counts per million
Deep deletion: Ddel
Deletion: Del
Norm: Normal copy number
Gain: Gain
Amp: Amplification
Log_2FC : Log_2(Fold Change)
P: P-value
-$Log_{10}P$: -Log_{10}(P-value)
PCC: Pearson Correlation Coefficient
SCC: Spearman Correlation Coefficient
DEG: Differentially Expressed Gene
DAC: Differentially Accessible Chromatin
FDR: False Discovery Rate (Benjamini-Hochberg)
QC: Quality Control
nuc: Nucleosome
TSS: Transcription Start Site
mtRNA: Mitochondial RNA
ALL: Acute Lymphoblastic Leukemia
ES: Enrichment Score
NES: Normalized Enrichment Score
oePBX1: overexpression vector PBX1
eVec: empty vector
GSEA: Gene Set Enrichment Analysis

SUPPLEMENTARY FIGURES

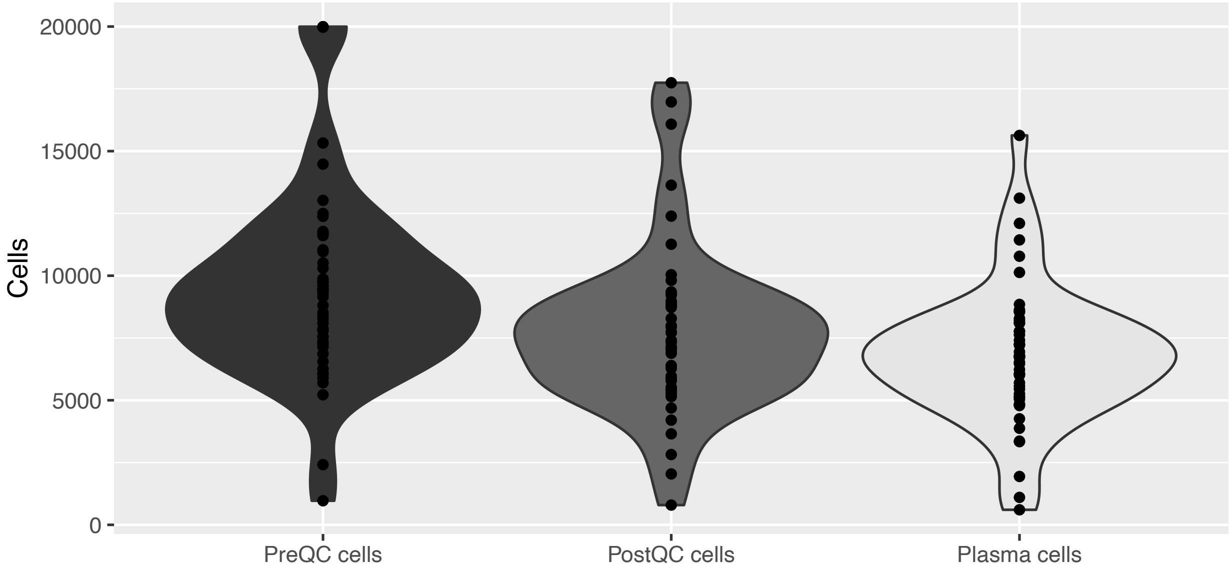

Supplementary Fig. 1 Cells per sample after each stage of quality control. PreQC cells denote any barcodes detected after sequencing with 10X Genomics. PostQC denotes cells after filtering out low quality cells. Plasma cells denotes cells after filtering out contaminant cell types from the microenvironment.

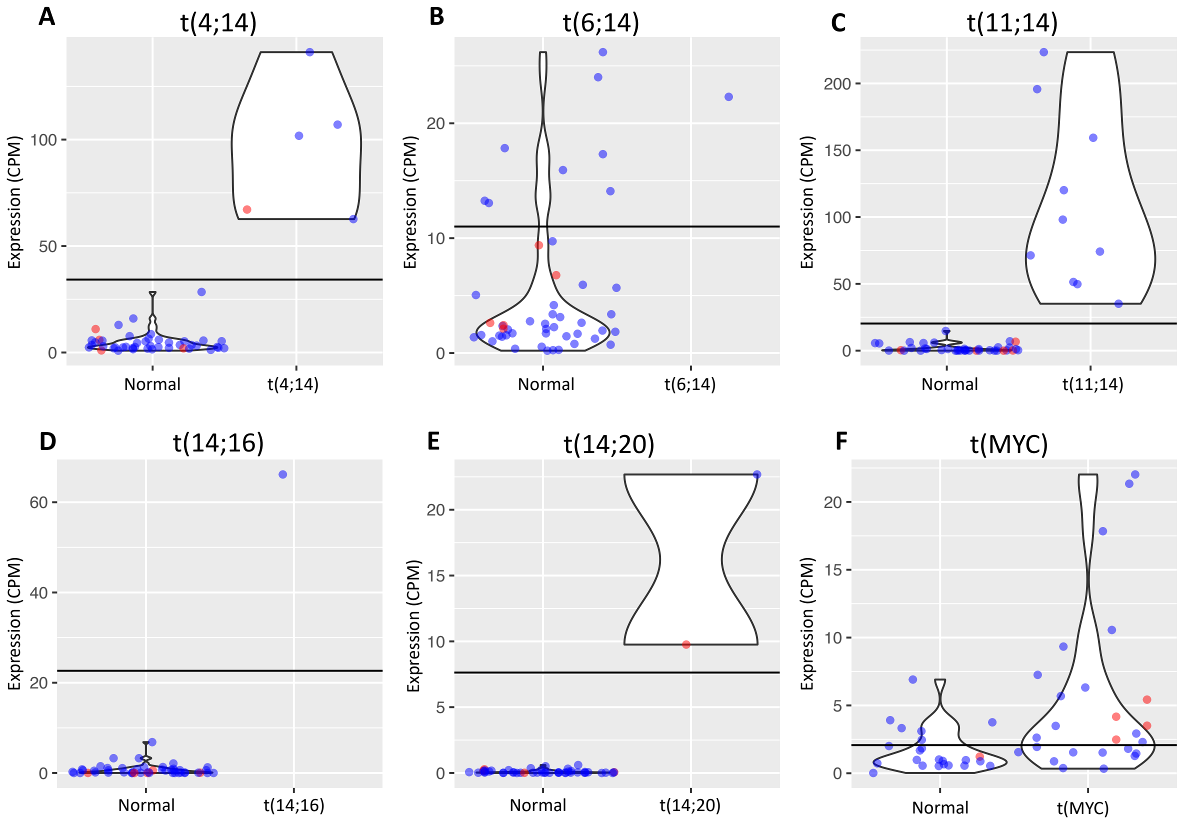

Supplementary Fig. 2 Imputation of translocations from canonical marker gene expression. The imputation was performed for **A)** t(4;14) (NSD2), **B)** t(6;14) (CCND3), **C)** t(11;14) (CCND1), **D)** t(14;16) (MAF), **E)** t(14;20 (MAFB), and **F)** t(MYC) (MYC). Note: blue dots: CNV status from WGS, and red dots: CNV imputed from canonical marker expression.

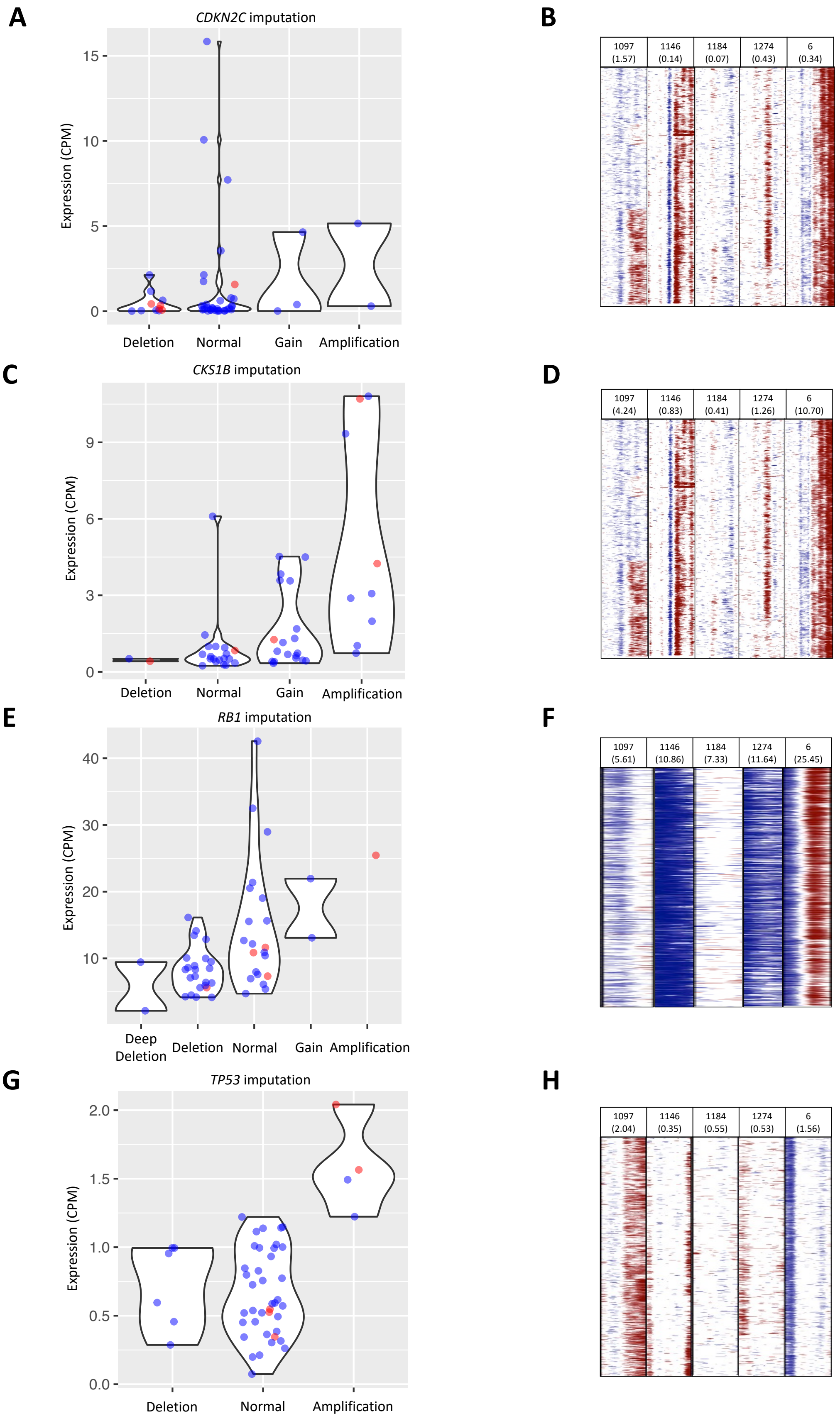
A
CDKN2C imputation
Expression (CPM)
0
5
10
15
Deletion
Normal
Gain
Amplification
B
1097 (1.57)
1146 (0.14)
1184 (0.07)
1274 (0.43)
6 (0.34)
C
CKS1B imputation
Expression (CPM)
0
3
6
9
Deletion
Normal
Gain
Amplification
D
1097 (4.24)
1146 (0.83)
1184 (0.41)
1274 (1.26)
6 (10.70)
E
RB1 imputation
Expression (CPM)
10
20
30
40
Deep Deletion
Deletion
Normal
Gain
Amplification
F
1097 (5.61)
1146 (10.86)
1184 (7.33)
1274 (11.64)
6 (25.45)
G
TP53 imputation
Expression (CPM)
0.0
0.5
1.0
1.5
2.0
Deletion
Normal
Amplification
H
1097 (2.04)
1146 (0.35)
1184 (0.55)
1274 (0.53)
6 (1.56)

Supplementary Fig. 3 Imputation of CNVs from canonical marker gene expression and inferred CNVs, 1p (**A,B**), 1q (**C,D**), 13q (**E,F**), and 17p (**G,H**). Marker expression, *CDKN2C* (**A**), *CKS1B* (**C**), *RB1* (**E**), and *TP53* (**G**), is plotted for each CNV group from WGS (blue dots: CNV status from WGS, and red dots: CNV status needed imputation). Inferred CNVs, 1p (**B**), 1q (**D**), 13q (**F**), and 17p (**H**) for each cell in each imputed patient based on inferCNV results. The mean expression in CPM of the marker genes are included in parentheses for *CDKN2C* (**B**), *CKS1B* (**D**), *RB1* (**F**), and *TP53* (**H**). Note: that the assignment of red dots in the marker gene plots (**A,C,E,G**) was overruled if the inferred CNV results were strong (**B,D,F,H**). Note: the correspondence to CNV copy number are, deep deletion (0), deletion (1), normal (2), gain (3), and amplification (≥ 4).

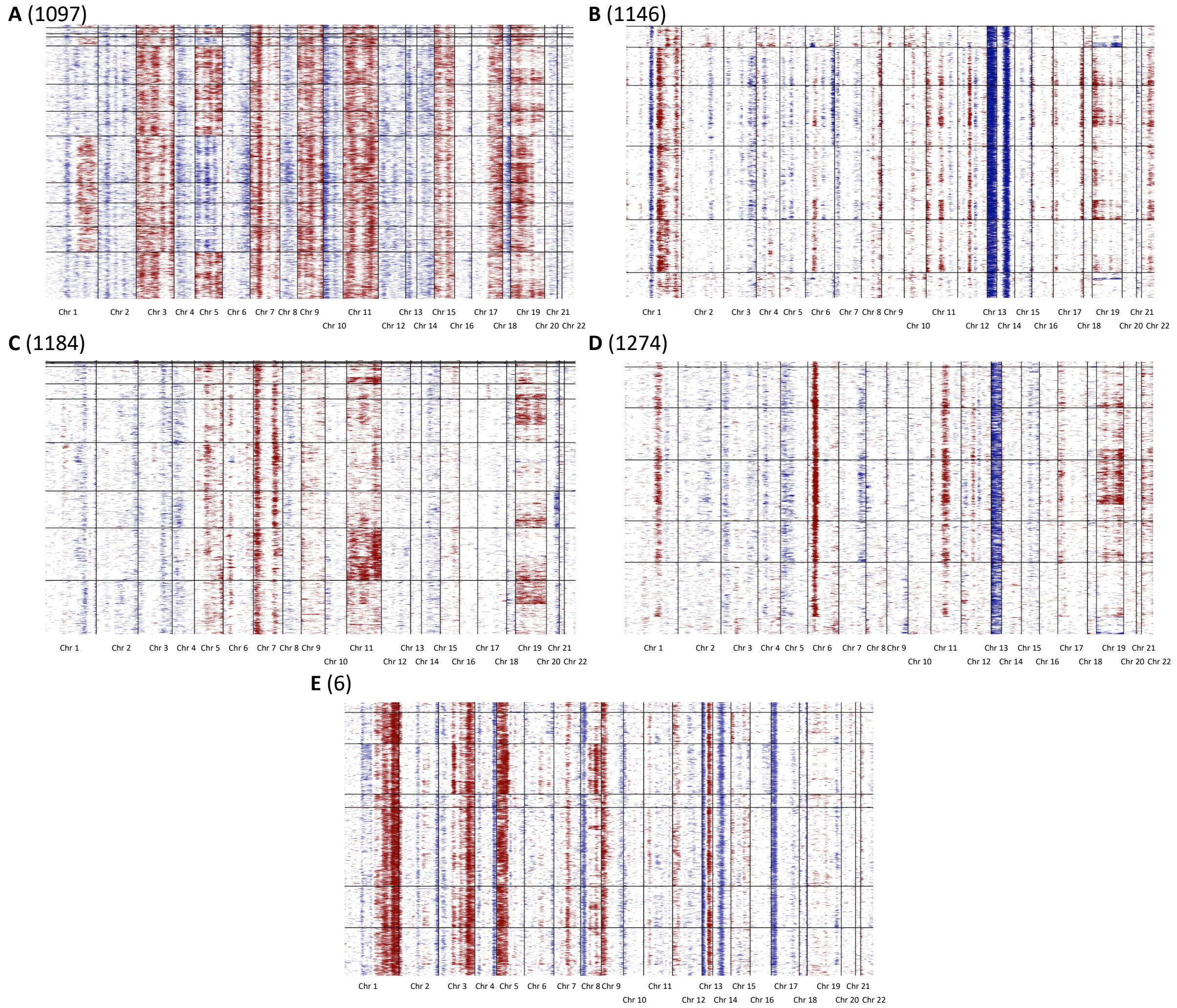

Supplementary Fig. 4 Imputation of HRD based on inferCNV results for samples 1097 **(A)**, 1146 **(B)**, 1184 **(C)**, 1274 **(D)**, 6 **(E)**.

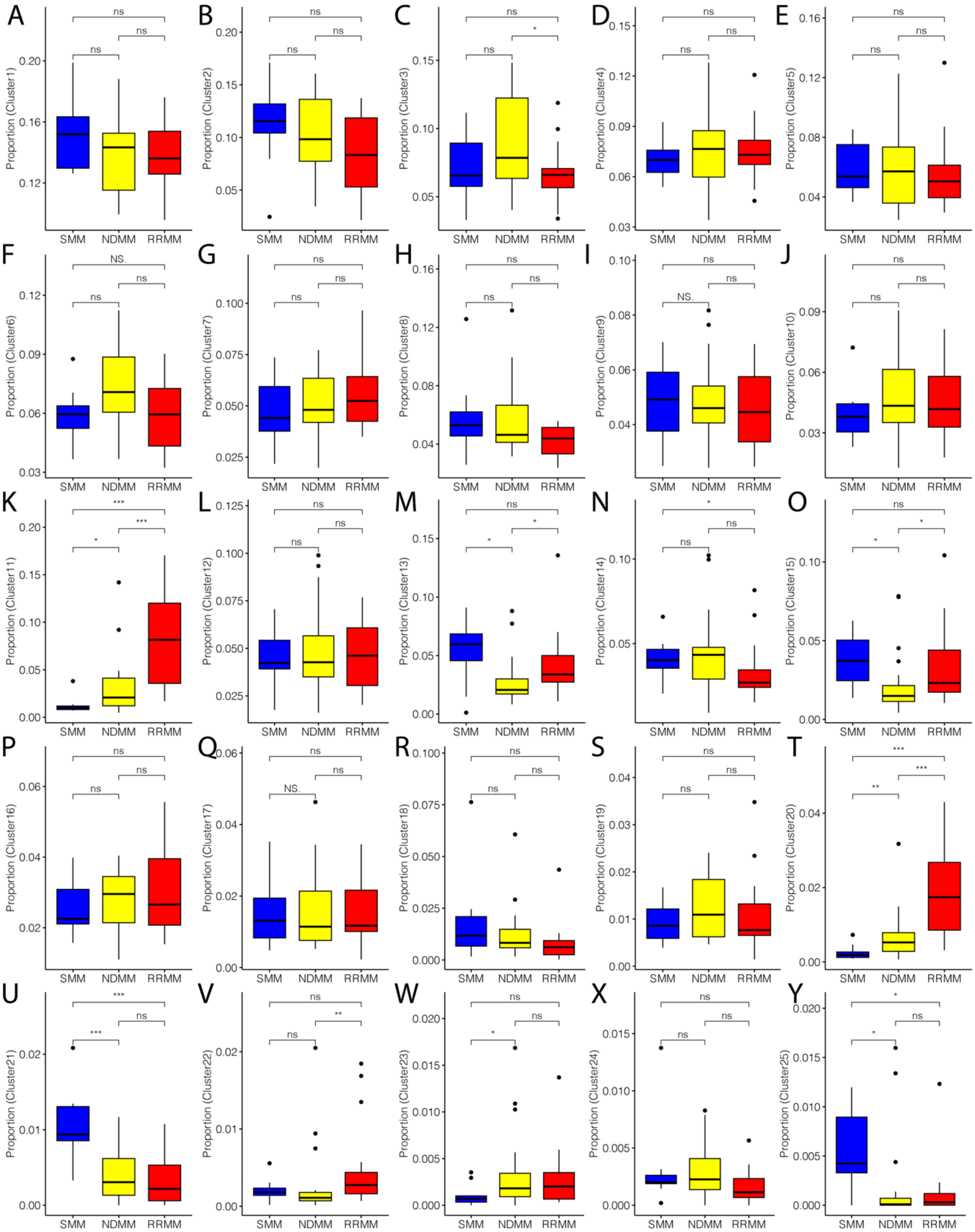

Supplementary Fig. 5 Proportion of each cluster stratified by stage of disease (SMM N=10, NDMM N=22, RRMM N=17): cluster 1 SMM=[0.1263,0.1296,0.1519,0.1638,0.1988], NDMM=[0.0994,0.1122,0.1433,0.1539,0.1881], RRMM=[0.0957,0.1258,0.1361,0.1539,0.1761] **(A)**, cluster 2 SMM=[0.0246,0.1029,0.1155,0.1331,0.171], NDMM=[0.0345,0.077,0.0983,0.138,0.1606], RRMM=[0.0215,0.0529,0.0833,0.1186,0.1373] **(B)**, cluster 3 SMM=[0.0329,0.0559,0.0655,0.0965,0.1116],

NDMM=[0.0404,0.0624,0.0785,0.1224,0.1482], RRMM=[0.034,0.0567,0.0661,0.0707,0.1188] (Wilcoxon test NDMMvRRMM P=4.19E-2) **(C)**, cluster 4 SMM=[0.054,0.0611,0.07,0.0766,0.0926], NDMM=[0.0341,0.0593,0.0765,0.0887,0.1279], RRMM=[0.0456,0.0673,0.0731,0.0817,0.1207] **(D)**, cluster 5 SMM=[0.0366,0.0458,0.0537,0.0801,0.0854], NDMM=[0.0245,0.0347,0.0571,0.0749,0.1227], RRMM=[0.0296,0.0395,0.0504,0.0613,0.13] **(E)**, cluster 6 SMM=[0.0366,0.0506,0.0595,0.0644,0.0876], NDMM=[0.0368,0.0605,0.0708,0.0894,0.1123], RRMM=[0.0323,0.0433,0.0594,0.0726,0.0902] **(F)**, cluster 7 SMM=[0.0218,0.0369,0.0441,0.0625,0.0736], NDMM=[0.0199,0.0417,0.0481,0.0658,0.0773], RRMM=[0.035,0.0425,0.0524,0.0643,0.0965] **(G)**, cluster 8 SMM=[0.0258,0.0454,0.053,0.0629,0.1257], NDMM=[0.0316,0.0412,0.0464,0.0689,0.1316], RRMM=[0.0238,0.0333,0.0439,0.0514,0.0557] **(H)**, cluster 9 SMM=[0.025,0.0371,0.0492,0.0615,0.0701], NDMM=[0.0243,0.0399,0.046,0.0544,0.0817], RRMM=[0.0246,0.0337,0.0446,0.0575,0.0694] **(I)**, cluster 10 SMM=[0.0231,0.0296,0.038,0.0451,0.0722], NDMM=[0.0127,0.0345,0.0434,0.0616,0.0907], RRMM=[0.0178,0.0328,0.0417,0.058,0.0813] **(J)**, cluster 11 SMM=[0.007,0.0081,0.0099,0.0119,0.0381], NDMM=[0.0049,0.0118,0.0208,0.0427,0.142], RRMM=[0.017,0.0357,0.0815,0.1201,0.1704] (Wilcoxon tests: SMMvNDMM P=3.10E-2, NDMMvRRMM P=1.95E-4, SMMvRRMM P=4.50E-6) **(K)**, cluster 12 SMM=[0.0176,0.0387,0.0423,0.0572,0.0706], NDMM=[0.0162,0.0349,0.0427,0.0577,0.0989], RRMM=[0.0203,0.0305,0.0461,0.0608,0.0768] **(L)**, cluster 13 SMM=[0.0012,0.0423,0.0596,0.0694,0.091], NDMM=[0.0083,0.0171,0.0206,0.0302,0.0881], RRMM=[0.0109,0.0272,0.0338,0.05,0.1356] (Wilcoxon tests: SMMvNDMM P=2.22E-2, NDMMvRRMM P=2.14E-2) **(M)**, cluster 14 SMM=[0.0204,0.034,0.0402,0.0474,0.0658], NDMM=[0.0092,0.0285,0.0434,0.0478,0.1022], RRMM=[0.0153,0.0241,0.0269,0.0345,0.0816] (Wilcoxon tests: SMMvRRMM P=4.59E-2) **(N)**, cluster 15 SMM=[0.0136,0.0245,0.0371,0.0524,0.0627], NDMM=[0.0043,0.0109,0.0149,0.0216,0.0783], RRMM=[0.0104,0.0173,0.0231,0.044,0.1042] (Wilcoxon tests: SMMvNDMM P=1.06E-2, NDMMvRRMM P=2.70E-2) **(O)**, cluster 16 SMM=[0.0157,0.0209,0.0225,0.0321,0.0398], NDMM=[0.011,0.0206,0.0295,0.0347,0.0404], RRMM=[0.0153,0.0207,0.0266,0.0395,0.0556] **(P)**, cluster 17 SMM=[0.0048,0.0071,0.0131,0.0201,0.0352], NDMM=[0.0052,0.0075,0.0114,0.0215,0.0463], RRMM=[0.0023,0.0101,0.0117,0.0216,0.0345] **(Q)**, cluster 18 SMM=[0.0017,0.0059,0.0117,0.022,0.0763], NDMM=[0.0017,0.0058,0.0083,0.0149,0.0607], RRMM=[3e-04,0.0025,0.0061,0.0093,0.0436] **(R)**, cluster 19 SMM=[0.0039,0.0054,0.0086,0.0129,0.0167], NDMM=[0.0047,0.0061,0.0109,0.0184,0.0241], RRMM=[0.0015,0.0065,0.0077,0.0132,0.0348] **(S)**, cluster 20 SMM=[9e-04,0.0012,0.0019,0.0029,0.0073], NDMM=[7e-04,0.0028,0.0053,0.008,0.0317], RRMM=[0.0032,0.0085,0.0174,0.0268,0.043] (Wilcoxon tests: SMMvNDMM P=4.00E-3, NDMMvRRMM P=5.45E-4, SMMvRRMM P=7.11E-6) **(T)**, cluster 21 SMM=[0.0033,0.0085,0.0094,0.0131,0.0208], NDMM=[0,0.0013,0.0031,0.0063,0.0117], RRMM=[0,6e-04,0.0022,0.0053,0.0108] (Wilcoxon tests: SMMvNDMM P=2.88E-4, SMMvRRMM P=5.74E-4) **(U)**, cluster 22 SMM=[2e-04,0.0013,0.0018,0.0024,0.0056], NDMM=[1e-04,7e-04,0.0011,0.0018,0.0205], RRMM=[7e-04,0.0016,0.0028,0.0044,0.0185] (Wilcoxon tests: NDMMvRRMM P=5.30E-3) **(V)**, cluster 23 SMM=[0,3e-04,7e-04,0.001,0.0035], NDMM=[0,9e-04,0.0018,0.0035,0.0169], RRMM=[3e-04,7e-04,0.002,0.0035,0.0137] (Wilcoxon tests: SMMvNDMM P=4.63E-2) **(W)**, cluster 24 SMM=[2e-04,0.0019,0.002,0.0026,0.0137], NDMM=[0,0.0014,0.0023,0.0041,0.0083], RRMM=[0,7e-04,0.0011,0.0023,0.0057] **(X)**, and cluster 25 SMM=[0,0.0033,0.0042,0.0093,0.012], NDMM=[0,0,1e-04,8e-04,0.016], RRMM=[0,0,3e-04,0.0012,0.0123] (Wilcoxon tests: SMMvNDMM P=1.55E-2, SMMvRRMM P=1.36E-2) **(Y)**. Note that the 5 number summaries are listed in brackets for each box in the following order [min, 25th percentile, median, 75th percentile, max].

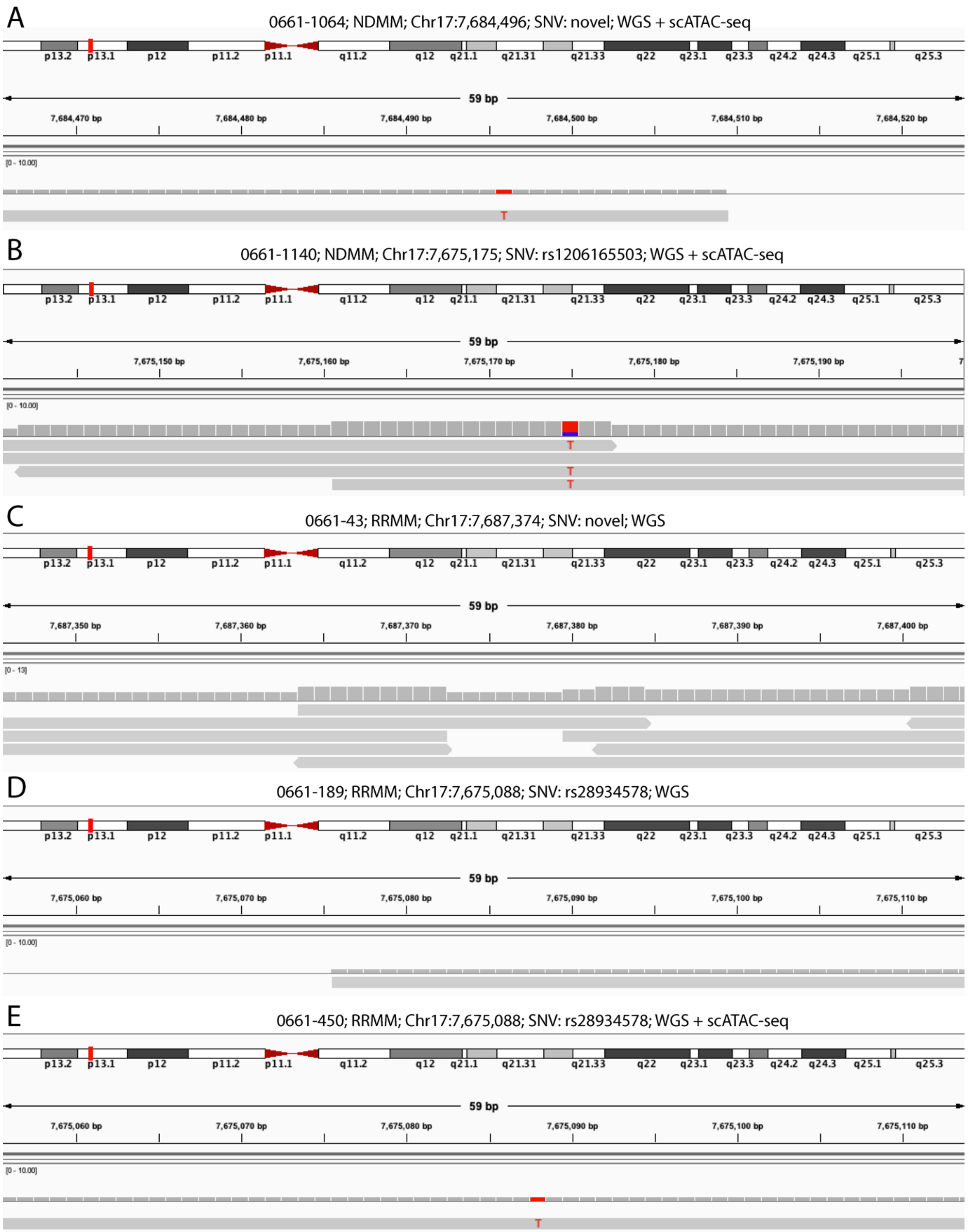

Supplementary Fig. 6 Variants identified in WGS with coverage in the $RRPC_{11}$ fraction of cells scATAC-seq data for patients: 0661-1064 **(A)**, 0661-1140 **(B)**, 0661-43 **(C)**, 0661-189 **(D)**, 0661-450 **(E)**.

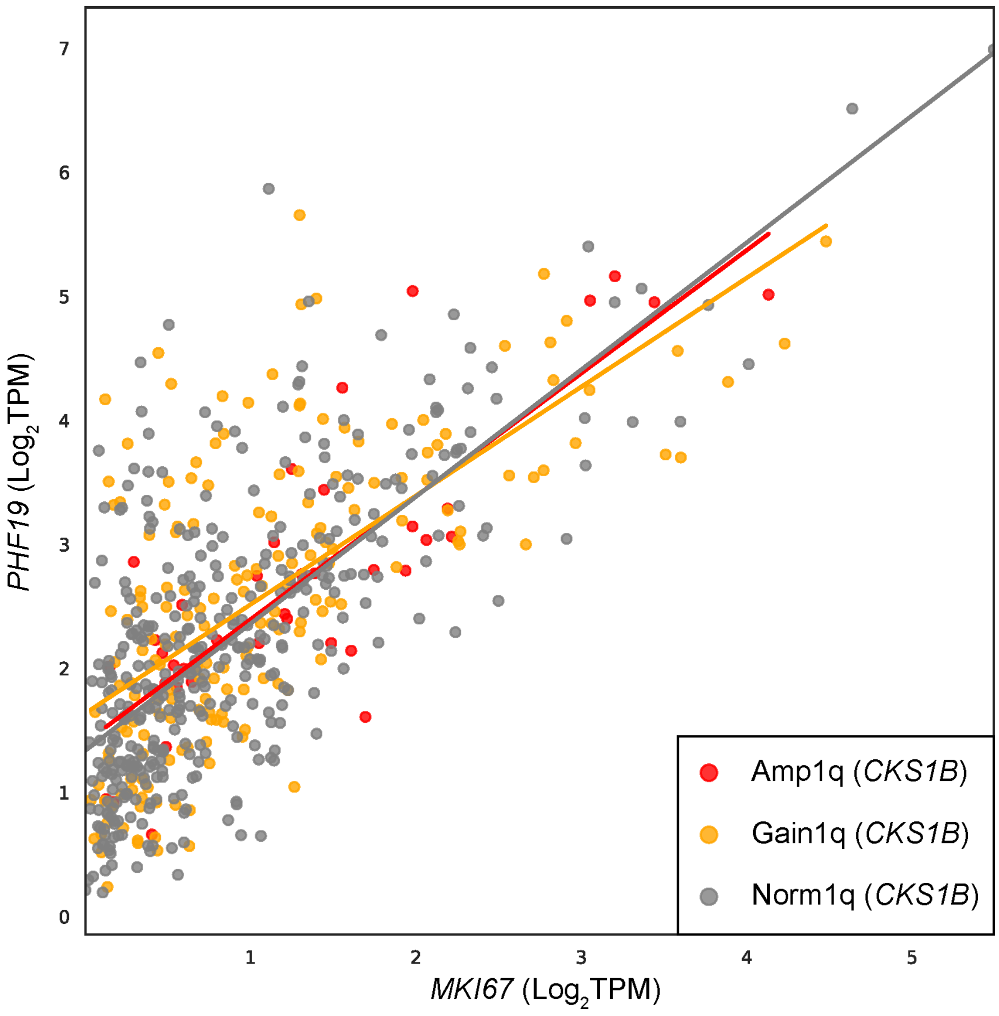

Supplementary Fig. 7 Association between MKI67 and PHF19 expression in the MMRF CoMMpass study cohort of patients. The points in the plot are labeled by their CNV status for 1q (*CKS1B*). The trend lines are fit for each individually.

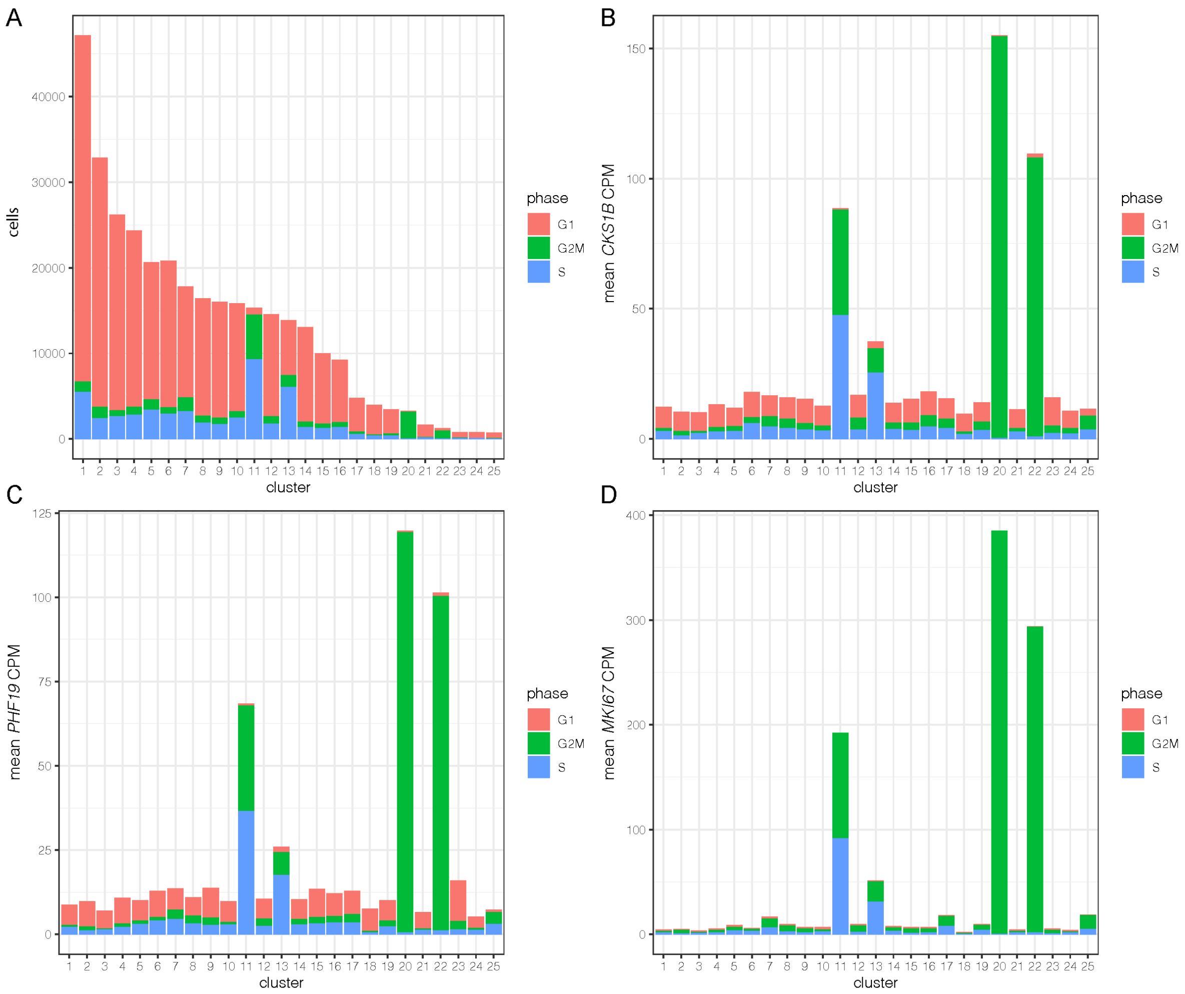

Supplementary Fig. 8 Cell cycle phase enrichment by cluster. **A)** Total number of cells in each cluster stratified into cell cycle phase. **B)** Average *CKS1B* (CPM) expression for each cluster stratified by the contribution of cells from each phase. **C)** Average *PHF19* (CPM) expression for each cluster stratified by the contribution of cells from each phase. **D)** Average *MKI67* (CPM) expression for each cluster stratified by the contribution of cells from each phase.

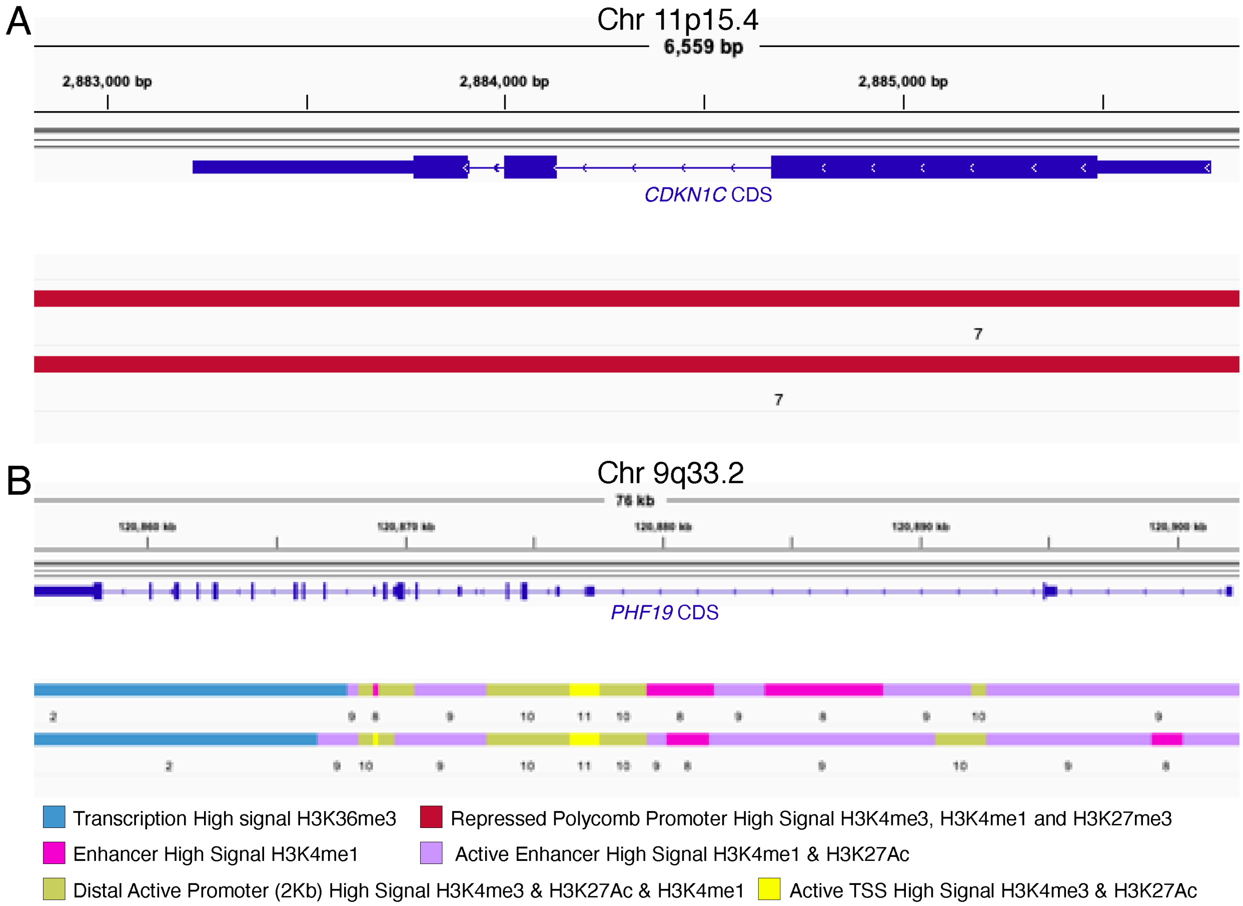

Supplementary Fig. 9 Regulatory regions determined from BLUEPRINT epigenomic data (GSE151556). **A**) CDKN1C has repressive regions associated with polycomb repressive complex 2 (H3K27me3). **B)** PHF19 has active regions representing a super-enhancer.

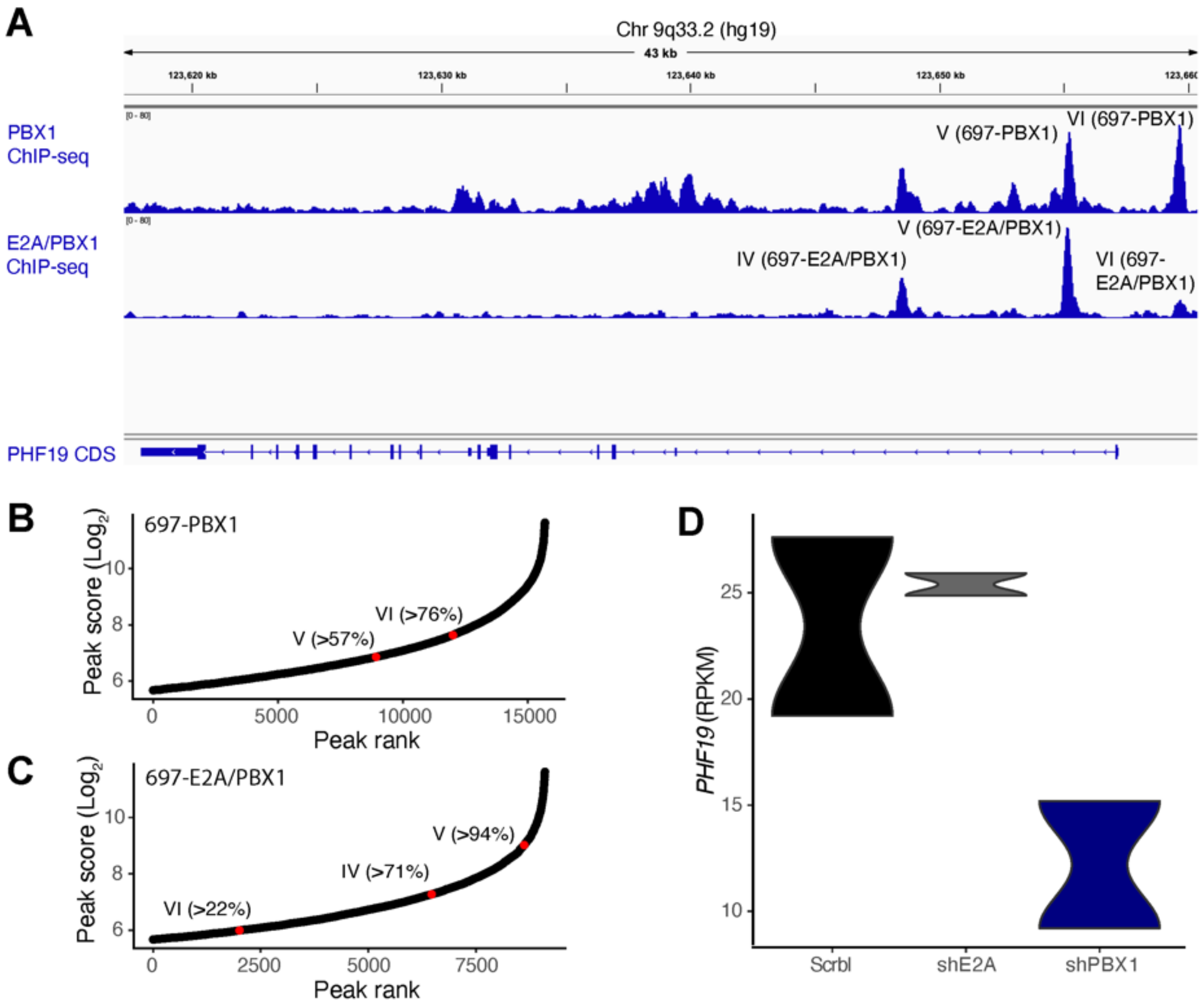

Supplementary Fig. 10 Association of the chromosome 1q TF, PBX1, with *PHF19* expression in pre-B cell ALL cell line 697 (GSE138031). **A)** PBX1 ChIP-seq peaks upstream of *PHF19* on chromosome 9. **B-C)** PBX1 **(B)** and E2A/PBX1 **(C)** ChIP-seq peak scores in pre-B ALL 697 cells with the percentile of each ChiP-seq peak marked. **D)** Differences in *PHF19* expression in pre-B ALL 697 cells treated with PBX1 or E2A shRNAs.

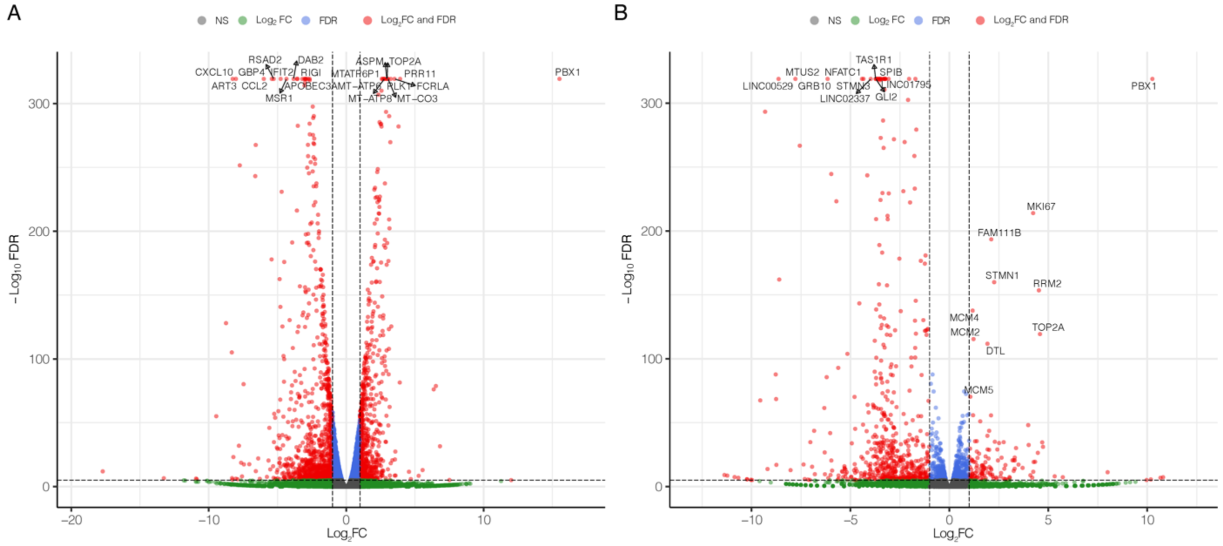

Supplementary Fig. 11 Differential expression results (Wilcoxon test) from oePBX1 compared to eVec in PCM6 **(A)** and MM1S **(B)** cell lines. Top 10 up- and down-regulated DEGs (based on FDR then $|Log_2FC|$) are labeled in the volcano plots. DEGs ($|Log_2FC| > 1$ and BH-FDR < 1e-5) are marked in red.

SUPPLEMENTARY TABLES

Supplementary Table 1 RNA QC metrics used for the preprocessing for the integration experiment and multiomic integration experiments.

Sample	max(RNA features)	min(RNA features)	max(mtRNA%)
Sample_0661_1053_5340777	4500	500	12
Sample_0661_1070_5347094	5000	500	10
Sample_0661_1127_5348553	4000	500	15
Sample_0661_1140_5350649	5500	500	20
Sample_0661_178_4153905	4000	500	20
Sample_0661_189_4829356	5000	500	10
Sample_0661_194_4156206	4500	500	10
Sample_0661_195_4172866	4000	500	15
Sample_0661_199_4680862	5500	500	20
Sample_0661_219_5167581	4500	500	15
Sample_0661_226_4678565	5500	500	10
Sample_0661_349_4156124	5000	500	10
Sample_0661_351_5350772	5000	500	15
Sample_0661_355_4680150	5500	500	10
Sample_0661_41_4308094	4500	500	8
Sample_0661_43_4046907	3500	500	15
Sample_0661_450_4408918	5000	500	20
Sample_0661_452_4306900	4500	500	8
Sample_0661_459_4312299	4500	500	10
Sample_0661_517_4835420	4000	500	12
Sample_0661_533_4835030	5000	500	12
Sample_0661_5_4680480	4000	500	15
Sample_0661_597_4677957	3500	500	20
Sample_0661_619_5690448	4500	500	15
Sample_0661_633_4447196	5000	500	20
Sample_0661_6_5961828	5500	500	15
Sample_0661_80_5965599	4500	500	15
Sample_0661_903_4835091	4500	500	12
Sample_0661_1008_4836785	5000	500	10
Sample_0661_1018_5339152	6000	500	15
Sample_0661_1021_5165969	4000	500	15
Sample_0661_1055_5340592	4000	500	8
Sample_0661_1059_5346970	5000	500	12
Sample_0661_1064_5346132	5000	500	15
Sample_0661_1092_5352736	4500	500	8
Sample_0661_455_4410289	2500	500	18
Sample_0661_634_4409412	4000	500	10
Sample_0661_1031_5167704	3800	500	10
Sample_0661_1034_5167024	4500	500	12
Sample_0661_1043_5167084	4500	500	18
Sample_0661_1066_5346256	4000	500	18
Sample_0661_1097_5357565	4500	500	20
Sample_0661_1132_5351658	3000	500	5
Sample_0661_1136_5352426	2500	500	12
Sample_0661_1146_5348025	4000	500	12
Sample_0661_1164_5698213	2000	500	12
Sample_0661_1184_5696042	3000	500	15
Sample_0661_1185_5696817	3000	500	8
Sample_0661_1274_5961619	3000	500	8

Supplementary Table 2 ATAC QC metrics used for the preprocessing of the multiomic integration experiments

Sample	max (ATAC features)	min (ATAC features)	max (nuc signal)	min (nuc signal)	max (TSS enrichmen)	min (TSS enrichment)
Sample_0661_533_4835030	10000	500	1.5	0.5	8	2
Sample_0661_189_4829356	5000	2000	1.2	0.7	8	3
Sample_0661_349_4156124	15000	500	1.3	0.8	8	3
Sample_0661_199_4680862	20000	500	1.5	0.7	7	3
Sample_0661_6_5961828	10000	500	1.8	0.6	8	3
Sample_0661_43_4046907	8000	500	0.8	0.3	8	2
Sample_0661_80_5965599	10000	500	1.2	0.6	7	4
Sample_0661_178_4153905	5000	500	2.2	1	5	2.5
Sample_0661_194_4156206	18000	500	1	0.5	7	3
Sample_0661_219_5167581	8000	500	1.3	0.6	8	3
Sample_0661_226_4678565	9000	500	1.2	0.6	8	4
Sample_0661_355_4680150	18000	500	1.5	0.8	6	3
Sample_0661_450_4408918	18000	500	1.4	0.7	7	3
Sample_0661_633_4447196	15000	500	1.4	0.5	7	3
Sample_0661_634_4409412	15000	500	1	0.3	6	3
Sample_0661_1018_5339152	15000	500	1.5	0.5	7	2
Sample_0661_1097_5357565	5000	500	2.2	0.8	7	1.5
Sample_0661_5_4680480	15000	500	1.2	0.7	7	3
Sample_0661_351_5350772	8000	500	1	0.4	7	3
Sample_0661_452_4306900	18000	500	1	0.5	7	3
Sample_0661_903_4835091	18000	500	1.2	0.7	6	3
Sample_0661_1008_4836785	18000	500	1	0.5	6	3
Sample_0661_1021_5165969	6000	500	3	1	7	1
Sample_0661_1034_5167024	12000	500	1.5	0.5	7	3
Sample_0661_1043_5167084	10000	500	1.5	0.5	8	3
Sample_0661_1053_5340777	15000	500	1	0.4	7	3
Sample_0661_1055_5340592	8000	500	2.3	0.8	6	2
Sample_0661_1059_5346970	15000	500	1.2	0.4	7	2
Sample_0661_1064_5346132	10000	500	1.7	0.7	8	2
Sample_0661_1066_5346256	11000	500	1	0.2	7	2
Sample_0661_1070_5347094	20000	500	1.3	0.7	5	2
Sample_0661_1092_5352736	12000	500	1.2	0.6	7	2
Sample_0661_1132_5351658	12000	500	1.2	0.5	7	3
Sample_0661_1136_5352426	12000	500	1.3	0.7	7	2
Sample_0661_1140_5350649	18000	500	1.5	0.5	6	3
Sample_0661_1146_5348025	12000	500	1	0.5	6	2
Sample_0661_1164_5698213	5000	500	2	0.8	6	2
Sample_0661_1184_5696042	10000	500	0.9	0.3	8	2
Sample_0661_1031_5167704	10000	500	1.5	0.6	7	2
Sample_0661_41_4308094	15000	500	1.8	0.8	5	3
Sample_0661_195_4172866	16000	500	1.3	0.5	7	2
Sample_0661_455_4410289	12000	500	1.5	0.7	9	3
Sample_0661_459_4312299	15000	500	1.5	0.7	7	2
Sample_0661_517_4835420	15000	500	1.6	0.7	6	3
Sample_0661_597_4677957	12000	500	1.3	0.7	8	2
Sample_0661_619_5690448	10000	500	1.2	0.4	8	2
Sample_0661_1127_5348553	20000	500	1.4	0.7	6	2
Sample_0661_1185_5696817	12000	500	1.3	0.5	8	3
Sample_0661_1274_5961619	10000	500	1.2	0.6	6	3

Supplementary Table 3 Imputation results for patients missing WGS. The notation in the table is as follows: translocation is not present (Neg), translocation is present (Pos), translocation is not calculated (NA), CNV copy number is 0 (Ddel), CNV copy number is 1 (Del), CNV copy number is 2 (Norm), CNV copy number is 3 (Gain), and CNV copy number is 4 (Amp).

	**t(4;14)**	**t(6;14)**	**t(11;14)**	**t(14;16)**	**t(14;20)**	**t(MYC)**	**1p**	**1q**	**13q**	**17p**	**HRD**
**0661-1097**	Neg	Neg	Neg	Neg	Neg	NA	Norm	Amp	Del	Norm	Pos
**0661-1146**	Neg	Neg	Neg	Neg	Pos	NA	Del	Gain	Del	Norm	Neg
**0661-1184**	Neg	Neg	Neg	Neg	Neg	NA	Norm	Del	Norm	Norm	Pos
**0661-1274**	Pos	Neg	Neg	Neg	Neg	NA	Norm	Gain	Del	Norm	Neg
**0661-6**	Neg	Neg	Neg	Neg	Neg	NA	Del	Amp	Amp	Del	Neg

Supplementary Table 4 High-risk genomic events evaluated in this project. Note: TENT5C is also frequently denoted FAM46C.

	Variables tested
**Translocations**	t(4;14), t(6;14), t(11;14), t(14;20), t(MYC)
**CNVs**	CDKN2C, RPL5, TENT5C, CKS1B, DNMT3A, CRBN, FGFR3, ADCY2, TNFAIP8, TNXB, PRKN, RAPGEF5, KLF14, DOCK5, MYC, CDKN2A, RNF20, TRAF2, RRAS2, CCND1, BIRC3, ATM, CDKN1B, BRCA2, RB1, DIS3, ABCD4, TRAF3, WDR72, BLM, CYLD, WWOX, MAF, TP53, AKAP1, ZNF426, ZNF227, SON
**Mutations**	KRAS, NRAS, TENT5C, DIS3, BRAF, TRAF3, TP53, CYLD, MAX, UBR5, USP7, IRF4, SP140, PTPN11, RB1, NFKBIA, RASA2, TGDS, CDKN1B, DUSP2, KLHL6, EGR1, FGFR3, ACTG1, ZNF292, HUWE1, CCND1, SAMHD1, ABCF1, CDKN2C, FUBP1, PRDM1, KMT2B, ATM, KMT2C, CREBBP, ARID1A, ATRX, NF1, TET2, KDM5C, ARID2, DNMT3A, KDM6A, MAFB
**Other**	HRD

Supplementary Table 5 VAF table for *TP53* variants in scATAC-seq reads. *TP53* variants identified by WGS were screened in the scATAC-seq split into the $RRPC_{11}$ cells and cells from all other clusters. For each patient with a *TP53* variant identified by WGS, the VAF was calculated for that variant in the $RRPC_{11}$ cells and the other cells combined. *Denotes calculates made in only patient sample where both $RRPC_{11}$ and Other cells had read coverage.

Patient	Location	Variant Class	Variant Type	Mutation	dbSNP RS	$RRPC_{11}$ VAF	Other cells VAF
0661-1064	Chr17:7,684,496	Intron	SNP	C->T	novel	100% (1/1)	60% (9/15)
0661-1140	Chr17:7,675,175	Nonsense	SNP	C->T	rs1206165503	75% (3/4)	20% (5/24)
0661-43	Chr17:7,687,374	Splice region	SNP	T->G	novel	0% (0/1)	67% (8/12)
0661-189	Chr17:7,675,088	Missense	SNP	C->T	rs28934578	0% (0/1)	67% (8/12)
0661-450	Chr17:7675088	Missense	SNP	C->T	rs28934578	100%	14% (1/7)
0661-41	Chr17:7,672,958	Intron	DEL		rs781465240		8% (1/13)
0661-178	Chr17: 7,673,609	Splice site	SNP	C->T	rs587781702		14% (2/14)
0661-349	Chr17: 7,674,291	Splice site	SNP	C->T	rs878854073		55% (5/9)
0661-349	Chr17:7,676,145-7,676,146	Frame shift	INS	->C	novel		13% (1/8)
0661-355	Chr17:7,663,150-7,663,151	Intron	INS	->TATT	rs370273514		
0661-597	Chr17: 7,669,977	Intron	SNP	C->T	rs1266557615		0% (0/5)
0661-1008	Chr17:7,674,902-7,674,919	In frame	DEL		novel		25% (2/8)
0661-1140	Chr17: 7674250	Missense	SNP	C->T	rs730882005		45% (5/11)
Mean						55%	32%
Median						75%	20%
Mean*						55%	32%
Median*						75%	20%

Supplementary Table 6 Association between *PHF19* and *MKI67* expression (Log_2TPM) in the MMRF CoMMpass cohort of NDMM patients (PCC and SCC).

Cohort	PCC	PCC P	FDR	SCC	SCC P	FDR
NDMM Amp1q	0.83	9.36E-11	9.36E-11	0.77	9.02E-09	9.02E-09
NDMM Gain1q	0.65	1.67E-23	2.23E-23	0.66	6.63E-24	8.84E-24
NDMM Norm 1q	0.70	9.11E-60	1.82E-59	0.65	9.22E-49	1.84E-48
NDMM (All patients)	0.70	3.99E-92	1.60E-91	0.67	2.02E-81	8.08E-81

Supplementary Table 7 Differential expression of MKI67 and PHF19 expression (Log_2TPM) in the MMRF CoMMpass cohort of NDMM patients (Wilcoxon test).

Gene	Group 1	Group 2	log2FC (Group1/Group2)	P	FDR
MKI67	Amp1q	Gain1q	0.27	2.78E-02	3.33E-02
MKI67	Amp1q	Norm1q	0.49	2.03E-04	6.08E-04
MKI67	Gain1q	Norm1q	0.21	9.76E-04	1.95E-03
PHF19	Amp1q	Gain1q	0.16	2.80E-01	2.80E-01
PHF19	Amp1q	Norm1q	0.52	2.42E-03	3.63E-03
PHF19	Gain1q	Norm1q	0.36	1.81E-04	6.08E-04

Supplementary Table 8 Cancer Hallmarks enriched (GSEA) for DEGs and DACs from $RRPC_{11}$.

CANCER HALLMARK	ES	NES	P	FDR	size	DEG type
E2F_TARGETS	0.70	3.16	0.00E+00	0.00E+00	188	RNA
G2M_CHECKPOINT	0.69	3.06	0.00E+00	0.00E+00	149	RNA
MITOTIC_SPINDLE	0.55	2.36	0.00E+00	0.00E+00	119	RNA
SPERMATOGENESIS	0.54	2.19	0.00E+00	0.00E+00	71	RNA
TNFA_SIGNALING_VIA_NFKB	-0.41	-2.70	0.00E+00	0.00E+00	76	RNA
TNFA_SIGNALING_VIA_NFKB	-0.63	-3.63	0.00E+00	0.00E+00	22	ATAC
KRAS_SIGNALING_UP	-0.51	-2.74	0.00E+00	3.16E-04	19	ATAC
HYPOXIA	-0.59	-2.46	0.00E+00	3.16E-03	11	ATAC
UV_RESPONSE_UP	-0.80	-2.22	8.63E-04	8.55E-03	5	ATAC
IL2_STAT5_SIGNALING	-0.39	-1.94	1.31E-02	3.20E-02	17	ATAC
ESTROGEN_RESPONSE_LATE	-0.34	-1.68	2.41E-02	8.04E-02	16	ATAC
OXIDATIVE_PHOSPHORYLATION	-0.23	-1.78	0.00E+00	8.29E-02	137	RNA
MYOGENESIS	-0.34	-1.70	1.78E-02	8.86E-02	16	ATAC
ESTROGEN_RESPONSE_EARLY	-0.33	-1.57	5.13E-02	1.17E-01	15	ATAC
DNA_REPAIR	0.36	1.53	1.12E-02	1.22E-01	99	RNA
PANCREAS_BETA_CELLS	-0.47	-1.53	6.30E-02	1.23E-01	7	ATAC
HYPOXIA	-0.22	-1.61	0.00E+00	1.62E-01	97	RNA
IL6_JAK_STAT3_SIGNALING	-0.42	-1.38	1.03E-01	1.89E-01	7	ATAC
INFLAMMATORY_RESPONSE	-0.29	-1.39	1.09E-01	2.01E-01	15	ATAC

Supplementary Table 9 Cancer Hallmarks enriched (GSEA) for DEGs and DACs from $RRPC_{20}$.

CANCER HALLMARK	ES	NES	P	FDR	size	DEG type
E2F_TARGETS	0.63	2.54	0.00E+00	0.00E+00	165	RNA
G2M_CHECKPOINT	0.69	2.76	0.00E+00	0.00E+00	146	RNA
HEME_METABOLISM	0.50	1.95	0.00E+00	1.06E-03	95	RNA
INTERFERON_ALPHA_RESPONSE	-0.22	-1.42	1.28E-02	2.41E-01	56	RNA
MITOTIC_SPINDLE	0.60	2.38	0.00E+00	0.00E+00	116	RNA
OXIDATIVE_PHOSPHORYLATION	-0.23	-1.72	0.00E+00	8.82E-02	108	RNA
SPERMATOGENESIS	0.52	1.94	0.00E+00	1.05E-03	63	RNA
TNFA_SIGNALING_VIA_NFKB	-0.34	-2.29	0.00E+00	4.77E-03	67	RNA
IL2_STAT5_SIGNALING	-0.44	-1.66	3.01E-02	1.19E-01	12	ATAC
INFLAMMATORY_RESPONSE	-0.43	-1.98	4.84E-03	2.59E-02	20	ATAC
TNFA_SIGNALING_VIA_NFKB	-0.62	-2.39	0.00E+00	1.53E-03	13	ATAC

Supplementary Table 10 Cancer Hallmarks enriched (GSEA) for DEGs and DACs from $RRPC_{22}$.

CANCER HALLMARK	ES	NES	P	FDR	size	DEG type
APICAL_SURFACE	-0.36	-1.36	1.11E-01	2.30E-01	16	RNA
COAGULATION	0.41	1.70	1.01E-02	2.42E-02	45	RNA
DNA_REPAIR	0.35	1.70	4.18E-03	2.56E-02	105	RNA
E2F_TARGETS	0.55	2.86	0.00E+00	0.00E+00	162	RNA
EPITHELIAL_MESENCHYMAL_TRANSITION	-0.26	-1.57	1.58E-02	8.58E-02	79	RNA
ESTROGEN_RESPONSE_EARLY	-0.34	-2.00	0.00E+00	1.28E-02	71	RNA
G2M_CHECKPOINT	0.65	3.34	0.00E+00	0.00E+00	142	RNA
GLYCOLYSIS	0.31	1.51	1.57E-02	9.11E-02	103	RNA
INFLAMMATORY_RESPONSE	-0.27	-1.57	2.00E-02	1.02E-01	69	RNA
INTERFERON_ALPHA_RESPONSE	0.35	1.57	1.82E-02	6.23E-02	64	RNA
MITOTIC_SPINDLE	0.44	2.20	0.00E+00	1.34E-04	120	RNA
MTORC1_SIGNALING	0.30	1.50	1.25E-02	9.13E-02	131	RNA
MYC_TARGETS_V1	0.35	1.84	0.00E+00	7.80E-03	169	RNA
MYC_TARGETS_V2	0.46	1.91	0.00E+00	4.49E-03	46	RNA
OXIDATIVE_PHOSPHORYLATION	0.35	1.79	0.00E+00	1.19E-02	159	RNA
SPERMATOGENESIS	0.53	2.31	0.00E+00	0.00E+00	56	RNA
TNFA_SIGNALING_VIA_NFKB	-0.31	-1.88	0.00E+00	1.98E-02	87	RNA
UV_RESPONSE_DN	-0.40	-2.47	0.00E+00	0.00E+00	78	RNA
DNA_REPAIR	0.65	2.70	0.00E+00	1.21E-03	15	ATAC
E2F_TARGETS	0.55	2.31	1.14E-03	4.15E-03	16	ATAC
G2M_CHECKPOINT	0.57	2.17	3.00E-03	7.13E-03	12	ATAC
INTERFERON_ALPHA_RESPONSE	0.51	2.12	1.10E-03	6.03E-03	16	ATAC
INTERFERON_GAMMA_RESPONSE	0.40	2.14	0.00E+00	6.59E-03	29	ATAC
MYC_TARGETS_V1	0.56	1.86	1.30E-02	2.95E-02	9	ATAC
OXIDATIVE_PHOSPHORYLATION	0.54	2.31	3.29E-03	6.03E-03	16	ATAC
UV_RESPONSE_DN	0.35	1.33	1.61E-01	2.48E-01	13	ATAC

Supplementary Table 11 Top 25 up- and down-regulated DEGs (EdgeR; based on FDR then $|Log_2FC|$) in PCM6 cell line for oePBX1 and eVec treated cells. The remaining significant DEGs at ($|Log_2FC|$ > 1 and BH-FDR < 1e-5) are can be found in **Supplementary Data 5**.

Gene	Log_2FC	P	FDR
PBX1	15.51	0.00E+00	0.00E+00
PRR11	3.92	0.00E+00	0.00E+00
FCRLA	3.51	0.00E+00	0.00E+00
PLK1	3.24	0.00E+00	0.00E+00
MT-CO3	3.04	0.00E+00	0.00E+00
TOP2A	2.97	0.00E+00	0.00E+00
ASPM	2.95	0.00E+00	0.00E+00
MT-ATP8	2.78	0.00E+00	0.00E+00
MT-ATP6	2.78	0.00E+00	0.00E+00
MTATP6P1	2.73	0.00E+00	0.00E+00
MT-CYB	2.66	0.00E+00	0.00E+00
MT-ND6	2.59	0.00E+00	0.00E+00
MT-ND4	2.57	9.30E-314	8.58E-311
MT-CO1	2.34	1.32E-310	1.18E-307
CENPF	2.90	3.73E-297	3.16E-294
GTSE1	3.12	9.92E-294	7.95E-291
MT-ND3	2.42	4.80E-289	3.66E-286
FOXM1	2.54	1.27E-287	9.22E-285
MT-ND5	2.49	1.90E-287	1.35E-284
NMU	3.80	1.39E-285	9.44E-283
SPAG5	2.57	1.48E-285	9.80E-283
COL24A1	3.21	3.03E-273	1.84E-270
NUSAP1	2.30	4.97E-252	2.44E-249
HMGB2	2.26	9.49E-250	4.52E-247
TK1	2.81	8.25E-241	3.70E-238
CXCL10	-8.26	0.00E+00	0.00E+00
ART3	-8.03	0.00E+00	0.00E+00
GBP4	-6.00	0.00E+00	0.00E+00
CCL2	-5.41	0.00E+00	0.00E+00
RSAD2	-5.27	0.00E+00	0.00E+00
IFIT2	-4.77	0.00E+00	0.00E+00
MSR1	-4.35	0.00E+00	0.00E+00
DAB2	-3.83	0.00E+00	0.00E+00
RIGI	-3.58	0.00E+00	0.00E+00
APOBEC3A	-3.55	0.00E+00	0.00E+00
TNFSF13B	-3.19	0.00E+00	0.00E+00
CCDC80	-3.04	0.00E+00	0.00E+00
TEX29	-2.99	0.00E+00	0.00E+00
CCDC146	-2.94	0.00E+00	0.00E+00
GBP1	-2.92	0.00E+00	0.00E+00
COL27A1	-2.77	0.00E+00	0.00E+00
OASL	-2.66	0.00E+00	0.00E+00
TNFSF10	-2.66	5.04E-322	5.12E-319
RSPO1	-2.99	2.45E-320	2.41E-317
FGL2	-3.03	6.46E-318	6.15E-315
LINC02574	-2.44	2.71E-301	2.36E-298
IFI6	-2.35	8.13E-294	6.69E-291
LINC00364	-2.40	2.53E-292	1.98E-289
RASGEF1B	-2.67	8.02E-288	5.96E-285
C3AR1	-3.52	3.93E-286	2.73E-283

Supplementary Table 12 Top 25 up- and down-regulated DEGs (EdgeR; based on FDR then $|Log_2FC|$) in MM1S cell line for oePBX1 and eVec treated cells. The remaining significant DEGs at ($|Log_2FC|$ > 1 and BH-FDR < 1e-5) are can be found in **Supplementary Data 6**.

	log2FC	PValue	FDR
PBX1	10.26	0.00E+00	0.00E+00
MKI67	4.23	1.79E-217	1.07E-214
FAM111B	2.12	6.53E-197	3.62E-194
STMN1	2.26	2.35E-163	1.14E-160
RRM2	4.52	5.91E-157	2.73E-154
MCM4	1.17	4.75E-141	2.04E-138
TOP2A	4.58	1.37E-122	4.80E-120
MCM2	1.22	1.13E-118	3.79E-116
DTL	1.92	5.29E-115	1.73E-112
MCM5	1.06	2.01E-73	4.71E-71
RASSF6	1.20	7.81E-59	1.54E-56
CDC6	2.11	9.02E-59	1.75E-56
E2F1	1.56	4.69E-54	8.51E-52
ASPM	3.98	4.34E-51	7.52E-49
UHRF1	2.01	1.41E-46	2.28E-44
CENPF	1.92	1.16E-42	1.75E-40
ZNF367	1.38	2.05E-38	2.79E-36
CDT1	1.30	2.57E-38	3.48E-36
SHCBP1	2.30	6.44E-38	8.54E-36
PCLAF	2.74	1.32E-37	1.74E-35
UBE2C	4.69	1.81E-37	2.35E-35
ZWINT	2.51	6.28E-37	7.98E-35
CCNB2	4.01	2.26E-35	2.69E-33
TROAP	4.73	6.21E-34	7.04E-32
MCM10	3.37	6.43E-33	7.10E-31
LINC00529	-8.63	0.00E+00	0.00E+00
MTUS2	-7.78	0.00E+00	0.00E+00
GRB10	-6.16	0.00E+00	0.00E+00
STMN3	-4.42	0.00E+00	0.00E+00
NFATC1	-4.34	0.00E+00	0.00E+00
LINC02337	-3.97	0.00E+00	0.00E+00
TAS1R1	-3.73	0.00E+00	0.00E+00
GLI2	-3.72	0.00E+00	0.00E+00
LINC01795	-3.65	0.00E+00	0.00E+00
SPIB	-3.65	0.00E+00	0.00E+00
LINC00276	-3.64	0.00E+00	0.00E+00
RSPO1	-3.61	0.00E+00	0.00E+00
GULP1	-3.56	0.00E+00	0.00E+00
LINC01090	-3.55	0.00E+00	0.00E+00
RIMBP2	-3.53	0.00E+00	0.00E+00
TEX29	-3.50	0.00E+00	0.00E+00
C19orf73	-3.48	0.00E+00	0.00E+00
SLC18A1	-3.47	0.00E+00	0.00E+00
SPANXA2-OT1	-3.38	0.00E+00	0.00E+00
RAD51B	-3.32	0.00E+00	0.00E+00
RALYL	-3.30	0.00E+00	0.00E+00
SLIT3	-3.28	0.00E+00	0.00E+00
OXCT2P1	-3.25	0.00E+00	0.00E+00
IGSF3P2	-3.24	0.00E+00	0.00E+00
OXCT2	-3.23	0.00E+00	0.00E+00
