# Supplementary material for: 1q amplification and PHF19 expressing high-risk cells are associated with relapsed/refractory multiple myeloma
Source: Nat Commun. 2024 May 16;15:4144. doi: 10.1038/s41467-024-48327-9 (PMC11099140; doi:10.1038/s41467-024-48327-9)
Supplement: Supplementary file 3 — Description of Additional Supplementary Files [file 41467_2024_48327_MOESM3_ESM.pdf]

Description of Additional Supplementary Files

File Name: Supplementary Data 1
Description: Significant associations (one-sided t-test) between cluster proportion and covariate status.

File Name: Supplementary Data 2
Description: Top 100 up-regulated and top 100 down-regulated DEGs (Wilcoxon test) for RRPC11.

File Name: Supplementary Data 3
Description: Top 100 up-regulated and top 100 down-regulated DEGs (Wilcoxon test) for RRPC20.

File Name: Supplementary Data 4
Description: Top 100 up-regulated and top 100 down-regulated DEGs (Wilcoxon test) for RRPC22.

File Name: Supplementary Data 5
Description: Significant (|Log2FC| > 1 and BH-FDR < 1e-5) up- and down-regulated DEGs (EdgeR) in PCM6 cell line for oePBX1 and eVec treated cells.

File Name: Supplementary Data 6
Description: Significant (|Log2FC| > 1 and BH-FDR < 1e-5) up- and down-regulated DEGs (EdgeR) in MM1S cell line for oePBX1 and eVec treated cells.
