# Supplementary material for: 1q amplification and PHF19 expressing high-risk cells are associated with relapsed/refractory multiple myeloma
Source: Nat Commun. 2024 May 16;15:4144. doi: 10.1038/s41467-024-48327-9 (PMC11099140; doi:10.1038/s41467-024-48327-9)
Supplement: Supplementary file 10 — Reporting Summary [file 41467_2024_48327_MOESM10_ESM.pdf]

nature portfolio

Corresponding author(s): Dr. Brian Walker

Last updated by author(s): Mar 15, 2024

Reporting Summary

Nature Portfolio wishes to improve the reproducibility of the work that we publish. This form provides structure for consistency and transparency in reporting. For further information on Nature Portfolio policies, see our Editorial Policies and the Editorial Policy Checklist.

Statistics

For all statistical analyses, confirm that the following items are present in the figure legend, table legend, main text, or Methods section.

n/a	Confirmed	
☐	☒	The exact sample size (*n*) for each experimental group/condition, given as a discrete number and unit of measurement
☐	☒	A statement on whether measurements were taken from distinct samples or whether the same sample was measured repeatedly
☐	☒	The statistical test(s) used AND whether they are one- or two-sided<br>*Only common tests should be described solely by name; describe more complex techniques in the Methods section.*
☐	☒	A description of all covariates tested
☐	☒	A description of any assumptions or corrections, such as tests of normality and adjustment for multiple comparisons
☐	☒	A full description of the statistical parameters including central tendency (e.g. means) or other basic estimates (e.g. regression coefficient) AND variation (e.g. standard deviation) or associated estimates of uncertainty (e.g. confidence intervals)
☐	☒	For null hypothesis testing, the test statistic (e.g. *F*, *t*, *r*) with confidence intervals, effect sizes, degrees of freedom and *P* value noted<br>*Give P values as exact values whenever suitable.*
☒	☐	For Bayesian analysis, information on the choice of priors and Markov chain Monte Carlo settings
☒	☐	For hierarchical and complex designs, identification of the appropriate level for tests and full reporting of outcomes
☐	☒	Estimates of effect sizes (e.g. Cohen's *d*, Pearson's *r*), indicating how they were calculated

Our web collection on statistics for biologists contains articles on many of the points above.

Software and code

Policy information about availability of computer code

Data collection	No software was used to collect data as the data were generated by sequencing patient samples.
Data analysis	The data analysis in this manuscript was conducted with publicly available software packages. The following command line tools were used in the study: mgp1000 (vNA), Conpair (v0.2), Strelka (v2.9.2), fpfilter (vNA), Variant Effect Predictor (v101), Manta (v1.6.0), ASCAT-NGS (vNA), cellranger-arc (v1.0.1), STAR (v2.7.11a), and featureCounts (v2.0.6). The following R packages were used in the study: Seurat (v4.3.0), Signac (v1.4.0), clusterProfiler (v4.0.5), inferCNV (v1.8.1), and CopyKAT (v1.0.8). The scripts used for the analysis in this manuscript can be found at the following GitHub site: https://github.com/tsteelejohnson91/MM_CD138pos_scripts.

For manuscripts utilizing custom algorithms or software that are central to the research but not yet described in published literature, software must be made available to editors and reviewers. We strongly encourage code deposition in a community repository (e.g. GitHub). See the Nature Portfolio guidelines for submitting code & software for further information.

Data

Policy information about availability of data

All manuscripts must include a data availability statement. This statement should provide the following information, where applicable:

- Accession codes, unique identifiers, or web links for publicly available datasets
- A description of any restrictions on data availability
- For clinical datasets or third party data, please ensure that the statement adheres to our policy

The single cell multiomic data, WGS data, and accompanying metadata have been deposited in the dbGAP database under accession code phs003220 (http://

www.ncbi.nlm.nih.gov/projects/gap/cgi-bin/study.cgi?study_id=phs003220.v2.p1). The single cell multiomic data and WGS data is available under controlled access and can be obtained by submitting a project request through dbGaP. Projects will be approved for data access if they are for research purposes and if the investigator is sponsored at an appropriate research institution. A response should be received after the request within two weeks and the data will be available for 12 months with optional renewal after approval. The processed single cell multiomic data has been deposited in the Synapse database under accession code syn52295155 (www.synapse.org/#!Synapse:syn52295155). The newly generated RNA-seq data have been deposited in the GEO database under accession code GSE254307 (https://www.ncbi.nlm.nih.gov/geo/query/acc.cgi?acc=GSE254307). The single cell multiomic, WGS, and RNA-seq datasets that were generated in this study were processed with GRCh38. The single cell multiomic data used the refdata-cellranger-arc-GRCh38-2020-A version while the RNA-seq data used the GRCh38 primary assembly with GENCODE comprehensive gene annotation for reference chromosomes (release 45). Publicly available RNA-seq and ChiP-seq datasets were retrieved from GEO (GSE16506011 and GSE13803122). The myeloma RNA-seq data used in the study was from the MMRF CoMMpass study IA18 (http://research.themmrf.org). The TF databases were downloaded from the following sources htTFtarget21 (http://bioinfo.life.hust.edu.cn/hTFtarget#!/download) and TF2DNA20 (https://www.fiserlab.org/tf2dna_db/downloads.html). The remaining data are available within the Article, Supplementary Information or Source Data files (https://doi.org/10.6084/m9.figshare.25563525).

Research involving human participants, their data, or biological material

Policy information about studies with human participants or human data. See also policy information about sex, gender (identity/presentation), and sexual orientation and race, ethnicity and racism.

Reporting on sex and gender	Self reported sex was used in this study. We included this as variable tested in our analysis.
Reporting on race, ethnicity, or other socially relevant groupings	Race was not included in this analysis due to low sample sizes.
Population characteristics	Genomic population characteristics were analyzed in the study, including: hyperdiploidy, copy number changes, translocations, and mutations. Furthermore, we included age as a covariate stratified between age greater than 65 years and age less than 65 years.
Recruitment	Recruitment of patients was conducted via the Indiana Myeloma Registry who recruits myeloma patients being treated at Indiana University Health. Biases in patient recruitment will stem from which patients are able to travel to Indiana University Health locations for treatment and which patients are able to donate the most aliquots from bone marrow aspirates.
Ethics oversight	Indiana University Human Research Protection Program Institutional Review Board

Note that full information on the approval of the study protocol must also be provided in the manuscript.

Field-specific reporting

Please select the one below that is the best fit for your research. If you are not sure, read the appropriate sections before making your selection.

☒ Life sciences ☐ Behavioural & social sciences ☐ Ecological, evolutionary & environmental sciences

For a reference copy of the document with all sections, see nature.com/documents/nr-reporting-summary-flat.pdf

Life sciences study design

All studies must disclose on these points even when the disclosure is negative.

Sample size	A sample size calculation for a two sample t-test was performed and we found that we can achieve a power of 0.80 given a Cohen"s D of 1.35, significance level of 0.05, and 10 samples per group. The number of samples was also dependent on the number of bone marrow aspirates available. Though with ten samples per group (our study: SMM=l0, NDMM=20, RRMM=l 7), we will still be able to detect differences especially when the effect size is large. For cellular level statistical testing, 335,000 cells have been profiled and small effect sizes will be detected.
Data exclusions	Pilot samples were excluded if they were not single cell multiomic data (N=2) or if they were a duplicate sample (N=l). No samples were excluded for sample quality issues.
Replication	The experiments described in this study were not replicated due to the number of bone marrow aspirates available and due to the cost performing single cell multiomics on a second validation set.
Randomization	Randomization is not relevant to our study design because there is no intervention. Covariates such as age and gender were tested to see if they were<br>associated with myeloma stages, cellular clusters, or genomic events.
Blinding	Blinding was not relevant in this study because there was no intervention. Furthermore, blinding would not be possible due to the sample identification process.

Reporting for specific materials, systems and methods

We require information from authors about some types of materials, experimental systems and methods used in many studies. Here, indicate whether each material, system or method listed is relevant to your study. If you are not sure if a list item applies to your research, read the appropriate section before selecting a response.

Materials & experimental systems

n/a	Involved in the study
x	Antibodies
	x Eukaryotic cell lines
x	Palaeontology and archaeology
x	Animals and other organisms
x	Clinical data
x	Dual use research of concern
x	Plants

Methods

n/a	Involved in the study
x	ChIP-seq
x	Flow cytometry
x	MRI-based neuroimaging

Eukaryotic cell lines

Policy information about cell lines and Sex and Gender in Research

Cell line source(s)	MM1S was from Walker lab. PCM6 was from Riken Catalogue #: RCB1460.
Authentication	For both these cell lines, targeted sequencing was performed and mutations and copy number changes were verified against data held elsewhere (https://www.keatslab.org/myeloma-cell-lines/hmcl-characteristics).
Mycoplasma contamination	All cell lines used in this study have tested negative for mycoplasma contamination. PCR based mycoplasma testing was performed with the Promokine PCR Mycoplasma test kit (catalog number PK-CA91-1024) and neither cell line tested positive against the included positive control.
Commonly misidentified lines (See ICLAC register)	None of the cell lines used in this study were in the ICLAC register of commonly misidentified cell lines version 12.

Plants

Seed stocks	*Report on the source of all seed stocks or other plant material used. If applicable, state the seed stock centre and catalogue number. If plant specimens were collected from the field, describe the collection location, date and sampling procedures.*
Novel plant genotypes	*Describe the methods by which all novel plant genotypes were produced. This includes those generated by transgenic approaches, gene editing, chemical/radiation-based mutagenesis and hybridization. For transgenic lines, describe the transformation method, the number of independent lines analyzed and the generation upon which experiments were performed. For gene-edited lines, describe the editor used, the endogenous sequence targeted for editing, the targeting guide RNA sequence (if applicable) and how the editor was applied.*
Authentication	*Describe any authentication procedures for each seed stock used or novel genotype generated. Describe any experiments used to assess the effect of a mutation and, where applicable, how potential secondary effects (e.g. second site T-DNA insertions, mosiacism, off-target gene editing) were examined.*
